# Smart Ungulates: What Sheep and Goats' Performances in a Reversed‐Reward Contingency Task Tell Us About the Evolution of Cognitive Flexibility

**DOI:** 10.1002/ece3.72506

**Published:** 2026-01-14

**Authors:** Laurie Castro, Raymond Nowak, Valérie Dufour

**Affiliations:** ^1^ Laboratoire de Psychologie Sociale et Cognitive (LAPSCO), CNRS Université Clermont‐Auvergne Clermont‐Ferrand France; ^2^ Physiologie de la Reproduction et Des Comportements (PRC), CNRS, IFCE, INRAE Université de Tours Nouzilly France

**Keywords:** cognitive flexibility, executive functions, inhibitory control, reversed‐reward contingency task, ungulates

## Abstract

Cognitive flexibility and inhibitory control are core executive functions that enable animals to adapt their behaviour to variable environments. Although these abilities are extensively studied in primates, and despite a growing interest in ungulate cognition, research specifically targeting executive functions in ungulates remains limited. In this study, we compare the performance of domestic goats ( *Capra hircus* ) and sheep ( *Ovis aries* ) in a reversed‐reward contingency (RRC) task. This task is traditionally used to test inhibitory control (the ability to resist a prepotent response), but it should also involve cognitive flexibility (the ability to adapt to changed contingencies). Overall, goats performed better than sheep. Two young goats met the success criterion spontaneously, and two more succeeded following corrective procedures. No sheep exceeded chance‐level performance. Only younger goats solved the task, confirming that cognitive flexibility is indeed a core process in this task for ungulates. The differences between sheep and goats reflect subtle differences in their behavioural flexibility both in the social and ecological domains. These results extend comparative cognition research beyond primates, highlighting ungulates as promising models for studying the evolution of executive functions.

## Graphical abstract

This study shows that young goats outperform sheep in the reversed‐reward contingency task, which is a cognitive test requiring both inhibitory control and cognitive flexibility. The results highlight the importance of cognitive flexibility when solving this type of task, and suggest that the differences between both species may reflect social and ecological behavioural flexibility. This highlights ungulates as promising models for studying the evolution of executive functions. Photo Credit: Maëlle Castro.

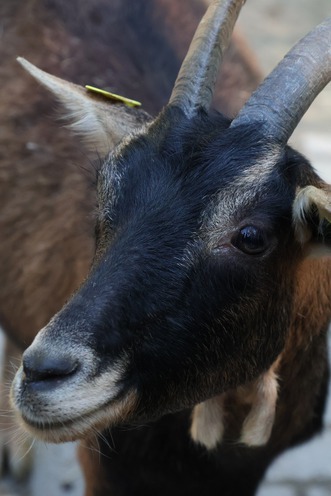

## 1 Introduction

Fitness and survival are linked to how individuals adapt to their physical and social environment (Völter et al. 2018). Cognitive flexibility and inhibitory control are two executive functions, which, along with working memory, reasoning or planning (Nigg 2017; Diamond 2013), enable animals to adjust rapidly to new situations. Animals demonstrating cognitive flexibility can pay attention to changes in different option contingencies and select the best option at a given time or in a given context. They can change their perspective to solve a problem (Diamond 2013; Highgate and Schenk 2021; Bonté et al. 2014). Cognitive flexibility also involves some inhibition. Inhibitory control is the ability to control attention, decisions and emotions to refrain from impulsive behaviours (Nigg 2017; Diamond 2013), and can help to solve problems, achieve future goals and optimise decisions.

Inhibitory control is traditionally investigated with the reversed‐reward contingency (RRC) task (Beran 2018; Boysen and Berntson 1995), which assesses animals' ability to inhibit their natural inclination to choose the larger of two amounts of food and instead select the smaller quantity to obtain the larger one. If they fail and choose the large reward, they receive the smaller one instead. Animals must show at least behavioural inhibition to refrain from taking the large reward, and even self‐control, which includes an effortful decisional component (Beran 2023). Furthermore, the task also requires cognitive flexibility, as individuals need to take into account the change in reward contingencies and modify their choice in order to select the small reward. The RRC task is not typically used as a test of cognitive flexibility. Instead, cognitive flexibility is often measured using the reversal learning task, in which subjects are trained to discriminate between two visual stimuli, one of which is positively reinforced (S+) and the other has to be avoided (S−). Once the contingencies are learned, a shift is implemented and S− is now rewarded (becomes S+) and vice versa (Highgate and Schenk 2021; Izquierdo et al. 2017). Compared to this reversal learning task, the RRC task appears to be emotionally salient because it involves choosing against spontaneous preferences rather than acquired preferences. It may involve both inhibition and cognitive flexibility, which are sometimes believed to be highly intertwined and interdependent (Chevalier and Blaye 2008).

Comparing cognitive flexibility and inhibitory control in several species is one way to investigate the evolution of cognition, and more particularly of executive functions. According to the social intelligence hypothesis and the ecological complexity hypothesis (Milton 1981; Dunbar 1998), species that live in complex societies with structured relationships, high levels of fission‐fusion dynamics, and/or in challenging ecological conditions, should have evolved advanced cognitive skills in order to cope with these challenges. They should perform better in an RRC task. Interestingly, for most species, the RRC task is difficult to solve. Some species are successful with a 1:4 food ratio between options, although success is achieved after many trials (great apes with a mean of 323 trials (Vlamings et al. 2006), mangabeys ~395 trials (Albiach‐Serrano et al. 2007), rhesus macaques ~1087 trials (Murray et al. 2005), sea lions ~123 trials (Genty and Roeder 2006)). Other species require corrective procedures, such as the large‐or‐none contingency procedure, where only the choice of the small quantity is rewarded (squirrel monkeys (Anderson et al. 2000), cottontop tamarins (Kralik 2005), black and brown lemurs (Genty et al. 2004), cleaner wrasse (Danisman et al. 2010), Japanese macaques (Silberberg and Fujita 1996)). Substituting the food items with some representation of the quantities also improves performance; when rewards are replaced by symbolic items (chimpanzees (Boysen et al. 1999)) or by tokens (capuchins (Addessi and Rossi 2010)), when food visibility is manipulated (great apes (Vlamings et al. 2006)), or when the dishes containing the rewards are coloured (Kralik et al. 2002). Some species, such as dogs, fail even when corrective procedures involving symbolic representations are applied (Fernand et al. 2018). Most species tested are primates, limiting the scope of the comparative approach.

Although ungulates, like primates, can live in large social groups with differentiated relationships and challenging environments, they are little studied for their cognitive flexibility, mainly due to the challenges of testing them in the wild. A recent study shows that domestic and wild ungulates living in fission‐fusion societies innovate more than other ungulates (Caicoya et al. 2023). Although selective breeding may recently have interfered with the natural evolution of species (Nawroth et al. 2022; Hare and Tomasello 2005), there is yet no clear evidence of a main effect of domestication on cognitive performances (Ferreira et al. 2023). More generally, domesticated species performances may be correlated to the same factors as wild species performances, such as brain size (e.g., shown to be positively related to behavioural inhibition (MacLean et al. 2014)), sociality (complex social systems may enhance behavioural inhibition and flexibility (Amici et al. 2008)) and/or foraging ecology (dietary diversity may enhance behavioural flexibility (Sol et al. 2002)). Thus, comparing the performance of domesticated ungulates could help to investigate the evolution of cognitive flexibility in non‐primate species. Individual factors such as personality (Coppens et al. 2010) or age (Dougherty and Guillette 2018) may also influence behavioural inhibition and cognitive flexibility. Inhibition appears to follow a U‐shaped relationship with age, with young and very old individuals showing greater impulsivity (Carlson et al. 2005; Doremus‐Fitzwater et al. 2012; Gratton et al. 2018). Conversely, older individuals appear to lack cognitive flexibility in reversal learning tasks (Lacreuse et al. 2018; Memisevic and Biscevic 2018; Gullstrand et al. 2022; Van Bourg et al. 2022). Bold, fast‐explorer individuals usually show lower flexibility and require more trials to reach the reversal criterion, unlike shy individuals (Bebus et al. 2016).

Here, we compare two phylogenetically close domestic ungulates, sheep ( *Ovis aries* ) and goats ( *Capra hircus* ), in the RRC task, to gain insight into the evolutionary processes of cognitive flexibility and inhibitory control in ungulates. Sheep and goats share a very similar history of evolution and domestication (Alberto et al. 2018). They differ specifically in their level of cohesion and feeding ecology, with goats living in more dispersed groups (Stanley and Dunbar 2013; Michelena et al. 2009) and being more food‐selective than sheep (Hosoi et al. 1995). Indeed, sheep (grazers) value proximity to their conspecifics more than patches of high‐quality food (Michelena et al. 2009; Dumont and Boissy 2000), while goats evolved as browsers and can spread over different patches. Several studies show that sheep and goats have impressive cognitive abilities. These include, for example, excellent long‐term memory (Kendrick et al. 2001; Briefer et al. 2012), social and asocial learning (Baciadonna et al. 2013; Nawroth et al. 2016; Castro et al. 2025), problem‐solving (Briefer et al. 2014), and causal reasoning abilities (Nawroth et al. 2014; Duffrene et al. 2022). Sheep and goats can also solve reversal learning tasks (Nawroth et al. 2022; Hunter et al. 2015). Both species have been suggested to differ in behavioural inhibition and flexibility in a detour task (Raoult et al. 2021), but their performances in the RRC task have not been compared to date. We predict that goats should perform better than sheep, because of their more flexible foraging ecology and/or sociality. We also investigate age‐related differences to help disentangle the contribution of inhibitory control and cognitive flexibility in the RRC task (Völter et al. 2018; Boogert et al. 2018; Bushby et al. 2018). A positive correlation between success and age would imply that the core process in this task is the control of impulsivity, whereas a negative correlation would suggest that cognitive flexibility is more central.

## 2 Methods

### 2.1 Subjects and Housing

**FIGURE A1:**
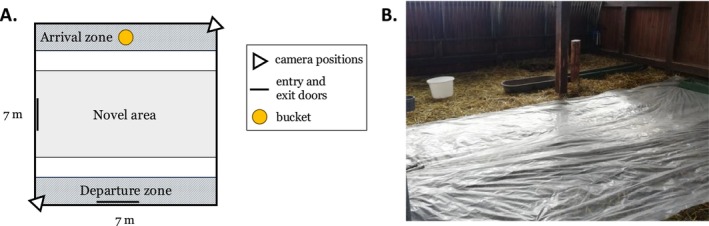
The main barn used for the Novel Area (NA) test. (A) Layout of the arena indicating the three zones, location of the novel area, location of doors, positioning of cameras and bucket; (B) Novel area used for the first session of tests.

**FIGURE A2:**
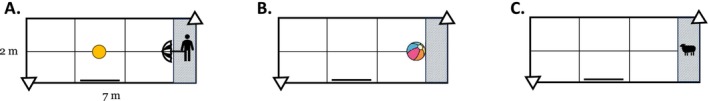
The test arena used for the Surprise (S) test, Novel Object (NO), Social Isolation (SI), Social Attraction (SA) and Social Retreat (SR) tests. The grey zone divided by a fence and was inaccessible to the test animal; it was used as the “umbrella zone” for the surprise test and the “conspecific zone” for the SA and SR tests. (A) Setup for the S test; The experimenter stood behind the fence and opened the umbrella. (B) Setup for the NO test; the NO was a multicoloured balloon in the shape of a birthday cake for the first session, and a blue rubber ball for the second session. (C) Setup for the SA and the first half of the SR test.

**FIGURE A3:**
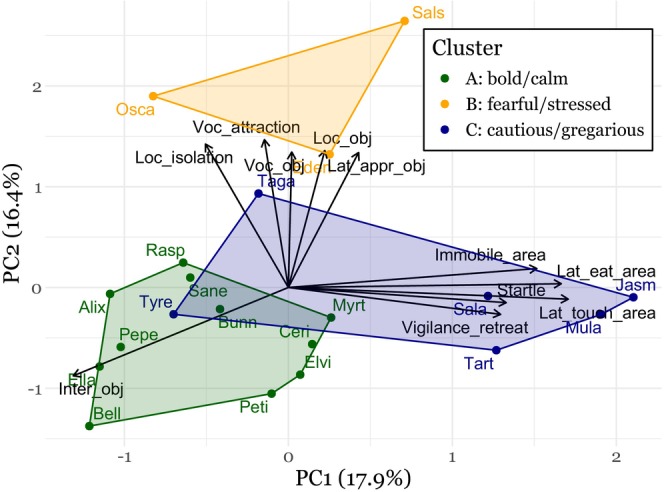
The cluster plot for the first test session of goats, showing the three clusters based on similarities and differences in the behavioural responses to all tests. Dimensions 1 and 2 approximate the first two principal components produced by a principal component analysis on the data. We showed the variables with loadings > 0.63 on the first and second principal components, as they were considered important in defining these dimensions.

**FIGURE A4:**
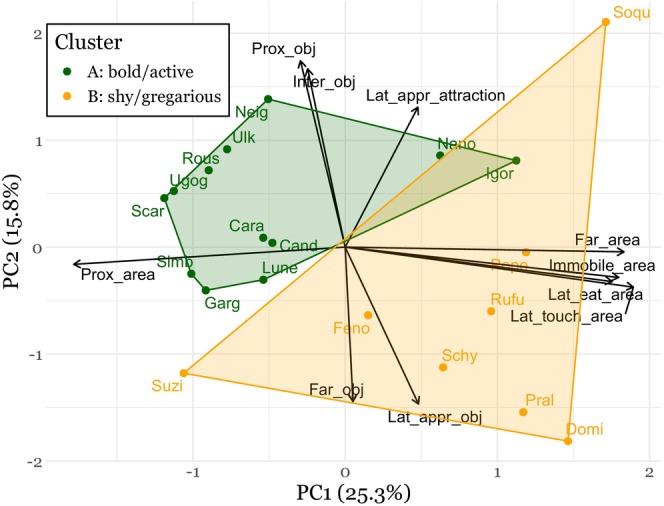
The cluster plot for the first test session of sheep, showing the two clusters based on similarities and differences in the behavioural responses to all tests. Dimensions 1 and 2 approximate the first two principal components produced by a principal component analysis on the data. We showed the variables with loadings > 0.63 on the first and second principal components, as they were considered important in defining these dimensions.

**FIGURE A5:**
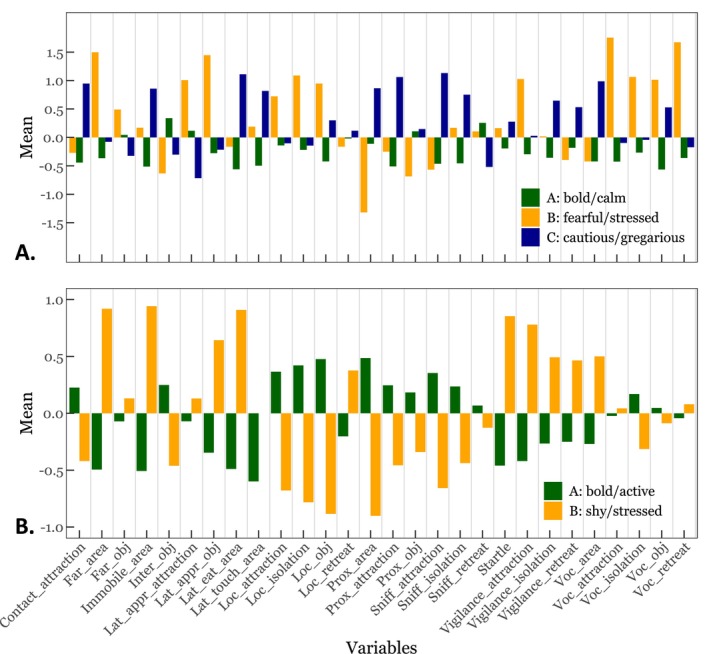
Cluster standardised means for each behavioural response, helping to determine the different personality profiles for (A) goats; (B) sheep.

The study group was composed of nineteen subjects (9 sheep and 10 goats, Table A1). All subjects were housed in the Friedel Park in Illkirch‐Graffenstaden (France), which is open to the public during weekends and school holidays. The animals share the same daily routine: they are free to roam in the two‐hectare area except for three hours in the morning when they are enclosed in the same barn for feeding. Individuals were trained/tested twice a day, with at least two hours between the sessions. Some (experienced) subjects had already participated in previous cognitive experiments involving the discrimination of different amounts of food. Based on personality tests conducted prior to this study (see Appendix 1 “Assessment of personality profiles” for all the details), we classified the subjects into different behavioural profiles (sheep: bold/active or shy/gregarious; goats: bold/calm, cautious/gregarious or fearful/stressed; Figure A3 and Figure A4). To this end, we measured the reactions of the subjects to a novel object, a novel area, a suddenness test (quick opening of an umbrella), social isolation and separation in a familiar context, and attraction towards familiar conspecifics (Table A2, Figure A1 and Figure A2). To assess the stability of personality over time, we repeated all tests for each individual. We quantified several behavioural parameters, such as locomotion, vigilance, vocalisations, latency to approach and interact with a novel object, area or with familiar conspecifics, or startle response during the surprise test (Table A3). We then performed a principal component analysis (PCA) on each species and each test session to identify groups of individuals exhibiting similar behaviours. We performed a k‐means clustering analysis on the principal components from each PCA, and we calculated the cluster standardised means for each behavioural response to define behavioural profiles (Figure A5).

### 2.2 Material and General Procedure

**FIGURE A6:**
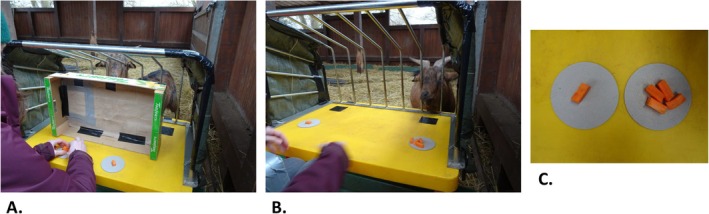
(A) Baiting out of sight of the subject; (B) Goat selecting a reward by indicating one side; (C) Carrot pieces used as rewards.

The experimental apparatus consisted of a table covered on both sides with grids to prevent access by the subject. Food rewards (identical pieces of carrots of 1 × 1 × 2cm) were placed on two grey plates covered by opaque overcups and spaced approximately 40 cm apart (Figure A6). A piece of cardboard was placed between the cups and the subject during the baiting. The subject was invited to stand in front of the centre of the table, then the experimenter removed the piece of cardboard and the overcups, revealing the two options. To “point” to the chosen option, the subject had to insert its snout through the grid and maintain the position. The experimenter then delivered the reward. From the final training step onward, the experimenter activated a hidden mechanism to bring the reward closer to the subject, rather than delivering it by hand.

### 2.3 Training

Naïve animals were first trained to choose between two options placed on the table, using the same procedure to which experienced individuals had been exposed (see Appendix 1 “Details about training”). Before starting the training, we familiarised the sheep and goats with the setup. Each animal was invited to enter the test area alone with the experimenter for a few minutes. It could then move around and explore the setup. Animals that showed any signs of distress, such as bleating, urinating, defecating or restlessness, after three days of habituation, were excluded from further training (one sheep). The first training step was designed to teach subjects to discriminate between two distinct locations on the table, of which only one was baited, and to correctly point to one option. We then conducted a second training step to test whether they could distinguish between one and four pieces of carrot. This training involved eight sessions per naïve individual, each consisting of 12 choices, with the side position of the larger quantity counterbalanced within a session and never presented more than three times in a row. Finally, the last training step aimed to check whether all subjects (experienced and naïve individuals) would prefer the larger quantity when presented with the two following ratios: 1 versus 4 and 1 versus 8. Each ratio was presented equally during 12‐trial sessions, and for each, the larger quantity was presented as many times on the right as on the left, but never more than three times in a row on the same side. Subjects completed a minimum of two and a maximum of 12 sessions. Training stopped once the success criterion was reached, i.e., 10 out of 12 successes during two consecutive sessions (corresponding to a significant binomial test), or 100% success in one session. One goat (ELV) did not reach this criterion and was excluded from the testing phase.

### 2.4 Testing

#### 2.4.1 Reversed‐Reward Contingency (RRC)

The testing general procedure was similar to that used in the last training period. Sheep and goats were tested in an RRC task using combinations of two amounts of food, 1 vs. 4 and 1 vs. 8. They performed 20 sessions each (except for one goat, EDE, who died before the end of the experiment and was therefore tested in only 12 sessions). The success criterion for a session was defined as 10 correct responses out of 12 trials, i.e., the subject selected the small reward in 10 trials. Additional sessions (max. 5) were conducted for subjects that did not reach success but achieved 50% of success (at least 7/12 correct responses) in at least one session. Once a subject reached the criterion, five more sessions were added to verify whether performance was maintained over time.

#### 2.4.2 Corrective Sessions: Large‐Or‐None Contingency

All subjects that did not learn to choose against their preference in the RRC test were given a large‐or‐none contingency test. This procedure consists of rewarding only the choice of the small reward. We only used the 1:4 ratio at this stage. If the subject selected array 1, it received array 4, but if it selected array 4, the experimenter removed array 1 from the plate and placed it visibly in front of the subject, showing the empty plate. The aim is to facilitate learning to choose the small reward, as selecting the large reward is not reinforced. Four sessions of 12 trials each (pseudo‐randomised for the side of the large reward) were administered per individual. As before, we added corrective sessions (max. 2) for subjects who did not reach the success criterion but reached 50% of success.

#### 2.4.3 Return to RRC

Once a subject had reached the criterion in a corrective session, it returned to the original RRC task for five 12‐trial sessions. Both ratios were again included to see if an individual who had learned to avoid the 4‐fold large reward was able to avoid the 8‐fold large reward.

### 2.5 Statistical Analyses

We examined the factors likely to influence success in the RRC task. Success (i.e., 1 if the subject chose the small reward and had then received the large reward; 0 otherwise) was analysed using a generalised linear mixed model (GLMM 1) with a binomial distribution and a logit link function (*lme4* package in R). Our predictors of interest were the species (sheep or goat), the ratio (1:4 or 1:8), the age of individuals (each individual was classified as young, up to 4 years old, adult, 5–7 years old, or aged, more than 8 years old), and their experience. Some subjects had previously participated in cognitive experiments involving quantity discrimination (experienced), while others had not (naïve). We included the number of the session (ranging from 1 to 20), any interaction between species and the other predictors as fixed factors, and subject identity as a random factor. We analysed the residuals to explore the fits of the model (*DHARMa* package in R) and calculated the variance inflation factors to assess multicollinearity (*car* package in R). Finally, we performed an analysis of variance to analyse the contribution of each factor and their interactions (*car* package), and post hoc analyses of main effects with Tukey correction (*emmeans* package). In addition, we conducted non‐parametric tests for each species to examine the effects of personality on success. We checked whether the personality profiles could predict the performance in the RRC test, as represented by the percentage of success in the first 20 sessions.

As several individuals showed a side bias (i.e., they chose the same side on at least 10 out of 12 trials during a session), we analysed how this side bias developed during the test. Following the same procedure as in the previous analysis, we ran a model (GLMM 2) with side bias as the response (1 if the individual had a bias during the session; 0 otherwise), and species, session, and the interaction between the two as predictors. We added the identity of the subject as a random factor.

## 3 Results

### 3.1 RRC Test

**FIGURE 1:**
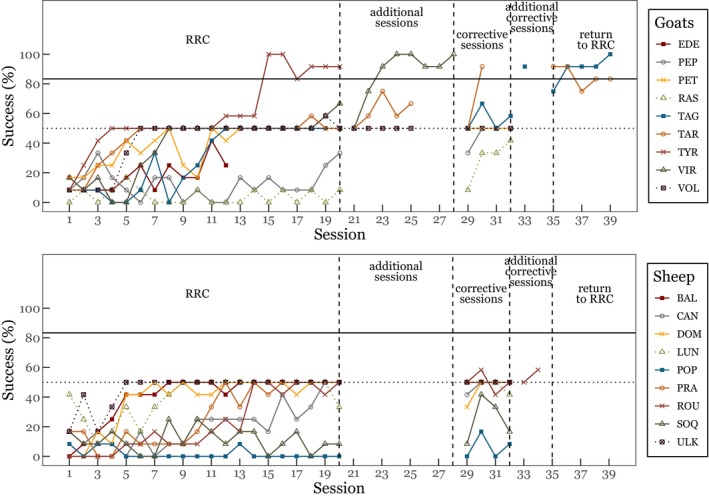
Subjects' performance across the experiment. The first step was the reversed‐reward contingency task (“RRC”), followed by “additional sessions” for subjects that had achieved more than 50% of success in at least one session (VIR, TAR and VOL). The next step was the “corrective sessions” (large‐or‐none contingency), followed by “additional corrective sessions” for subjects that had reached more than 50% of success in at least one corrective session, which was the case for TAG and ROU. Finally, subjects that succeeded in the corrective sessions were given a further five sessions without correction (“return to RRC”; TAG and TAR). The success per session indicates the percentage of times the subject selects the small reward; The horizontal dotted line shows the chance level (50% success), whereas the horizontal black line shows the success criterion (at least 10/12 correct responses).

**FIGURE 2:**
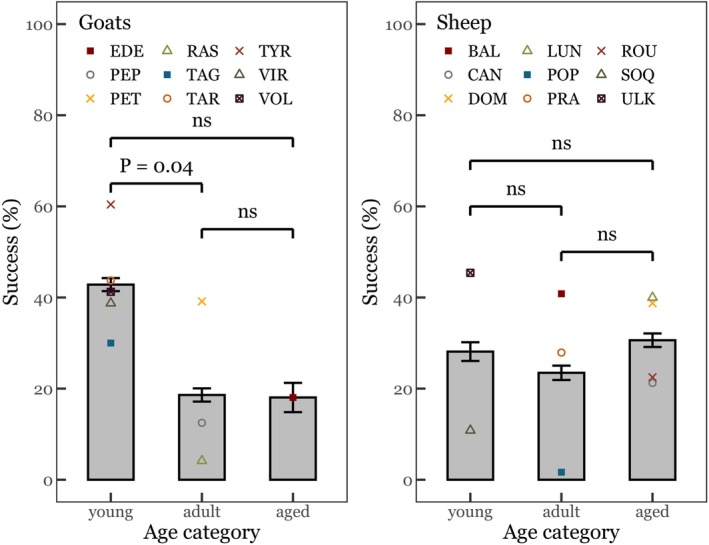
Effect of age on performance (± SE) in the RRC (reversed‐reward contingency) task (left: Goats; right: Sheep).

**FIGURE A7:**
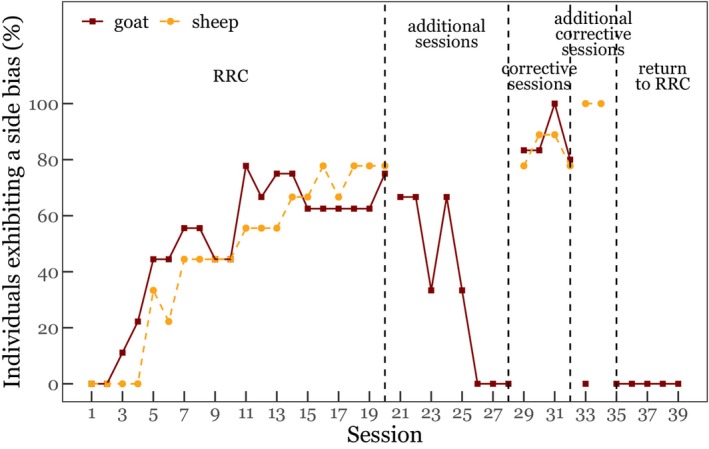
Percentage of subjects exhibiting a side bias (at least 10/12 choices on the same side) over the course of the experiment.

One male goat (TYR) reached the success criterion (at least 10/12 successful trials) in session 15 and then maintained its performance in the next five sessions. During the first 20 sessions (RRC task), no sheep and almost no goat exceeded 50% of success (Figure 1) and most individuals significantly selected the large reward (Table A4). Two subjects (sheep ULK and goat TAR) chose equally often each option (Table A4). Performances of all individuals improved over the 20 sessions (GLMM 1, see Appendix 1 “Detailed results”, Table A5). Young goats achieved higher success than adults, but this was not the case for sheep (Figure 2). Experience in previous quantity discrimination tasks had no effect on success. Finally, sheep tended to be more successful for the 1:4 ratio than for the 1:8 ratio (Table A6). The Kruskal‐Wallis tests showed no significant effect of personality profiles on success. Seven goats (out of 9) and 7 sheep (out of 9) developed side preferences. Both species progressively exhibited a side bias during the first 20 sessions, leading them to reach the chance level (50% of success, GLMM 2, Table A7). However, sheep seemed to show a steep increase in side bias over time, whereas goats exhibited a much more gradual change (Figure A7).

Three young goats (TAR, VIR and VOL) exceeded 50% of success (without reaching the success criterion) in at least one session and were then given additional sessions. One of these goats (VIR) achieved the success criterion in its third additional session and maintained it in the next five sessions.

### 3.2 Corrective Sessions

All individuals (except the goats TYR and VIR that spontaneously succeeded in the RRC task) were further tested in corrective sessions. During these sessions, only two goats (TAR and TAG) were successful and moved to the next phase. The other subjects maintained a preference for one side if they already had one, or developed a side bias, leading them to achieve a mean success that was not different from chance. One exception to this is one sheep (POP), which kept on mostly selecting the large reward (mean success rate: 6.3%).

### 3.3 Return to RRC

The two goats (TAG and TAR) that succeeded in the corrective sessions were then given five more sessions without correction and were able to maintain their success, achieving respectively 90% and 85% success (Figures 1 and 2).

## 4 Discussion

Two young goats spontaneously solved the RRC task. Two others succeeded after a correction procedure. Success in the youngest goats only suggests that, in addition to inhibition, cognitive flexibility is at work in this task. Sheep performed no better than chance, raising questions about this difference with goats.

Succeeding in the RRC task is not a trivial feat. Animals must point to what they don't want, probably making the task ecologically non‐significant (Shifferman 2009). Many species struggle to avoid the large reward in order to obtain it (Boysen and Berntson 1995; Murray et al. 2005; Danisman et al. 2010). Notably, young goats that succeed without a modified procedure do so in fewer trials than most of the primates (168 trials for TYR and 264 for VIR), making their performance comparable to that of non‐human primates. Only two goats succeeded in this task spontaneously (and two after corrections), which matches the proportion of individuals that are successful in other species (e.g., one successful chimpanzee out of four; Boysen et al. 1999). Not all goats may be able to solve this task, but we show that this capacity is within the reach of the species and can be expressed under the right conditions. A larger sample size is needed to investigate this question further. The correction procedure helped some individuals to achieve 50% success or to overcome their bias (TAG and TAR). The RRC task is difficult because subjects are still rewarded for selecting the large quantity (whereas they are not when they select the “wrong” stimulus in a traditional reversal learning task). Small reinforcement changes can lead to failures or successes in maximising choice (Silberberg and Fujita 1996), which would explain in part improvement in performance with large‐or‐none contingency. Sea lions show similar impressive performance (they can solve the task in ~123 trials). However, they had to press levers to make their choice, rather than responding directly to the food items which may be easier to do compared to a direct choice (Beran 2018; Genty and Roeder 2006). In our study, sheep and goats point directly to the food with their snout, similar to natural foraging situations (Zobel et al. 2019). Refraining from reaching for the largest amount requires inhibition as carrots are highly valued in their diet.

In contrast to goats, sheep did not exceed chance level, revealing marked interspecific differences. This questions whether this difference in performance can be explained by divergent skills in executive functions. First, Vlamings et al. (2006) suggested that poor performances in the RRC task may be linked to previous intensive training on other numerical tasks. However, we found no difference in task‐solving ability between naïve and experienced individuals, in either species. In addition, subjects must show some form of inhibition to refrain from the response to reach for the large quantity (Shifferman 2009). We must distinguish between simple behavioural (motor) inhibition and true self‐control (as defined by (Beran 2015), see also (Dunbar and Shultz 2025)), which implies a decisional and strategic component. According to Dunbar and Shultz (Dunbar and Shultz 2025), true self‐control is better assessed with tasks such as the A‐not‐B or delayed gratification paradigms, and may only be found in anthropoid primates. However, ungulates can indeed succeed in such paradigms (goats in the A‐not‐B task (Nawroth et al. 2015) and horses in a delayed gratification task (Brucks et al. 2022)). Thus, there is no reason to consider that only anthropoid primates should show self‐control. According to Beran (2018), the RRC task could be used to evaluate self‐control as opposed to behavioural inhibition. Our study does show that sheep and goats refrained from “always selecting the large reward”, but we cannot conclude about the type of inhibition involved. Our data will undoubtedly contribute to a larger comparative investigation of this question. Murray et al. (2005) suggest that animals can solve the task at a 50% success rate because they make random choices. But solving the task with 100% success requires persistence in the detection of errors, leading to a change of strategy. Sheep and older goats typically reach the 50% step, mostly thanks to a side bias, also documented in other species (Albiach‐Serrano et al. 2007; Anderson et al. 2000; Danisman et al. 2010). Only younger goats persist in detecting errors. This would hint at a stronger cognitive flexibility in (younger) goats than in sheep. Other studies also show that the best performers in the RRC task were the younger subjects (Albiach‐Serrano et al. 2007; Genty et al. 2004). In primates, cognitive flexibility declines with age, which is associated with changes in neuroanatomy and neurochemistry in the frontal lobes (Lacreuse et al. 2018; Kupis et al. 2021). Difficulties in overcoming a bias and reframing a choice situation are also related to aging in humans (Wilson et al. 2018).

Sheep and goats are respectively grazers and browsers, which eat food evenly distributed, which has led to the idea that they lack inhibitory control (MacLean et al. 2014; Stevens et al. 2005). Since both species successfully refrained from “always selecting the larger reward”, there is no evidence that goats strongly differ from sheep in their behavioural inhibition. We found no effect of personality on success. It is also unlikely that sheep and goats differed in their reactivity to social isolation during the task, given that we observed no signs of distress or lack of motivation to participate and eat the rewards. The difference between the two species is likely due to differences in cognitive flexibility, reflecting their social and ecological behaviour. Indeed, sheep (grazers) prefer to feed on more evenly distributed resources (high‐fibre herbaceous species) than goats (browsers) (Papachristou et al. 2005; Yiakoulaki et al. 2009), which prefer to forage on low‐fibre but rarer vegetation and are more selective. In addition, sheep are thought to live in more cohesive groups than goats (Michelena et al. 2009), which exhibit higher levels of fission‐fusion (Stanley and Dunbar 2013). Goats appear to have a complex and differentiated relationship with their conspecifics, whereas sheep mainly gather based on their social activity and when disturbed (Miranda‐de La Lama et al. 2019; Sibbald and Hooper 2004; Pitcher et al. 2017). Sociality and group living can strongly impact performance, as more cohesive species like sheep are thought to lack behavioural flexibility (Amici et al. 2008). The less flexible foraging and social strategy of sheep could correlate with their lower cognitive flexibility in the RRC task (Amici et al. 2008; Hosoi et al. 1995; Raoult et al. 2021; Rosati 2017; Bartolome et al. 1998). Similar correlations between behavioural and cognitive flexibility have been reported in other species (Tomasek et al. 2024; Mikhalevich et al. 2017). Testing other ungulates with contrasting social and/or foraging flexibility could help disentangle the effects of each variable on the evolution of cognitive flexibility.

This is the first evidence that individuals of a domesticated species can solve this challenging task, which several species struggle with. Although popular belief considers ruminants not to be very smart, some species like goats can show quite impressive cognitive abilities (Grimm 2023), and other ungulate species with similar behavioural flexibility may show similar performances in an RRC task. More generally, we advocate for using a comparative approach to investigate other executive functions such as memory, planning, and reasoning (Duffrene et al. 2022) in ungulates and identify the factors involved in the evolution of their cognition.

## Author Contributions

**Laurie Castro:** data curation (equal), formal analysis (equal), investigation (equal), methodology (equal), writing – original draft (equal), writing – review and editing (equal). **Raymond Nowak:** validation (equal), writing – review and editing (equal). **Valérie Dufour:** investigation (equal), methodology (equal), project administration (lead), supervision (lead), validation (lead), writing – review and editing (lead).

## Funding

The authors did not receive financial support from any organisation for the submitted work.

## Ethics Statement

All the procedures were behavioural and non‐invasive, and subjects could choose to stop participating at any time. The project was reviewed and approved by the appropriate local research ethics committee (CEEA, Comité d'Éthique pour l'Expérimentation Animale du Val de Loire, n °ce19‐2023‐0504‐1).

## Conflicts of Interest

The authors declare no conflicts of interest.

## Supporting information


**Video S1:** A goat solving the reversed‐reward contingency task.

## Data Availability

The data set used and analysed in this study can be found in the Supporting Information.

## Appendix 1

### 1.1 Details About Subjects and Housing

The animals were housed in the Friedel Park in Illkirch‐Graffenstaden, France. Sheep and goats lived together with ponies, alpacas, pigs, chickens, cows, and donkeys in a two‐hectare area that was open to the public during weekends and school holidays. Water was available ad libitum. Both sheep and goats shared the same daily routine: they had access to the same barn for sleeping and were fed together in the morning for approximately three hours in the same enclosed barn with hay, straw, and wheat flour. They had access to food enrichment, including fresh fruit, vegetables, and twigs at various times of the day. Not all of the animals were born in the park, but they live there until the end of their natural lives. Considering the unbalanced sample size towards some breeds (e.g., more Alpine goats), we lack the statistical power to investigate potential differences in personality profiles or performances between breeds.

**TABLE A1:** Characteristics of the subjects participating in the experiment (ELV was excluded as it did not meet the success criterion during training). Initial learning performance relates to the second training step, in which subjects had to choose between one and four food rewards (eight 12‐trial sessions). The number of training sessions refers to the third training step, which aimed to establish whether the subjects preferred to select the larger quantity (success criterion: Achieving 10 out of 12 successes in two consecutive sessions or achieving 100% success in one session).

Species	Subject	Breed	Experience	Age	Sex	Initial learning performance	Training sessions	Personality profile
Goat	EDE	Alpine	Yes	10	F	Medium (74.2%)	2	Fearful – Stressed
	ELV	Poitevine	No	10	M	Bad (61.2%)	12	Bold – Calm
	PEP	Alpine	Yes	6	F	Medium (77.3%)	3	Bold – Calm
	PET	Dwarf	Yes	6	M	Medium (78.9%)	5	Bold – Calm
	RAS	Lorraine	Yes	5	M	Good (81.2%)	2	Bold – Calm
	TAG	Alpine	Yes	3	F	Bad (60.4%)	5	Cautious – Gregarious
	TAR	Alpine	Yes	3	F	Bad (66.7%)	4	Cautious – Gregarious
	TYR	Alpine	Yes	3	M	Medium (74%)	2	Cautious – Gregarious
	VIR	Saanen (crossbred)	No	1	M	Bad (63.5%)	4	
	VOL	Saanen (crossbred)	No	1	M	Good (79.2%)	6	
Sheep	BAL	Texel × Suffolk	No	5	F	Medium (77.1%)	2	
	CAN	Suffolk	No	8	F	Medium (75%)	6	Bold – Active
	DOM	Ouessant	Yes	11	M	Bad (67.4%)	11	Shy – Gregarious
	LUN	Crossbred	No	10	F	Bad (62.5%)	2	Bold – Active
	POP	Suffolk	Yes	6	M	Good (84.2%)	2	Shy – Gregarious
	PRA	Suffolk	Yes	6	F	Good (81.2%)	4	Shy – Gregarious
	ROU	Mourerous	No	9	F	Good (81.2%)	5	Bold – Active
	SOQ	Texel	Yes	4	F	Good (79.2%)	3	Shy – Gregarious
	ULK	Friesian milk	No	2	M	Bad (70.8%)	7	Bold – Active

### 1.2 Assessment of Personality Profiles

As previous research has shown that personality in sheep and goats is multidimensional (Atkinson et al. 2022), we exposed individuals to different situations, stimuli and contexts. Our objective was to define behavioural profiles based on the various expressions of these traits. To this end, we measured the reactions of 20 goats and 20 sheep to a novel object, a novel area, a suddenness test, isolation and separation in a familiar context, and attraction towards familiar conspecifics.

The personality tests took place in the main barn, which measured 7 × 7 m, where the animals were accustomed to being fed each morning. This enabled individuals to be isolated from the main group's view. Except for the novel area test, the main barn was divided into two using a large grey screen (1.5 m high) to create a test arena measuring 6 × 2 m. This test arena was divided into six equal sections using white markings on the ground (Figure A2). This division was used to evaluate the area crossed by the subject during the novel object test, the social isolation test, the social attraction test and the social separation test. The location of the animal was noted continuously enabling us to measure the “number of sections entered” by the animal, which was an indicator of locomotion during the test. Two video cameras were mounted on opposing walls of the arena to record all tests continuously. These video recordings were later analysed to quantify several behavioural parameters during the different tests (Table A3).

The individuals were familiarised with the test arena and then tested, following the steps described in Table A2. For each individual, each step was conducted on a different day and the tests were always conducted the day after the familiarisation step. The first session of tests was conducted from 29 January to 15 April 2024. Five months after the first session (from 21 June to 24 September 2024), we repeated all steps for each individual (except for one sheep, IGO, who died between the two sessions), in order to assess the stability of personality over time. The animals were always free to move and were never led by a halter. They voluntarily entered each test arena for every step of the testing process.

**TABLE A2:** Order of the steps followed by each individual for the personality testing. These steps were repeated five months later for the second session.

Steps	Familiarisation	Personality test	Description
1	Habituation to voluntarily entering the barn and eating		No signs of distress; the animal crosses the barn and eats from the bucket within 30 s in 7/8 trials over 3 consecutive sessions.
2		Novel Area (NA)	The animal has to cross a novel area to eat within 5 min
3	Habituation to the new test arena		No signs of distress; the animal eats from the bucket within 30 s for 3 consecutive sessions.
4		Surprise test (S)	Quick opening of an umbrella.
5	Habituation to the marks on the ground		No signs of distress or unusual reactions to the marks; The animal eats in the bucket within 30 s.
6		Novel Object (NO)	A novel object is placed in a familiar environment for 3 min
7		Social Isolation (SI)	The animal is isolated from its conspecifics with no possibility of joining them for 3 min
8		Social Attraction (SA)	The animal is placed near a familiar conspecific and is allowed to join it for 3 min
9		Social Retreat (SR)	The animal is near a familiar conspecific in the test arena, and then the conspecific is removed for 90 s.

#### 1.2.1 Novel Area (NA) Test

##### 1.2.1.1 Habituation to Eat

A habituation session consisted of training the animal to voluntarily enter the main barn, cross it and eat from the bucket located opposite the door. The main barn was divided into three sections: a starting zone close to the door, a central zone and an arrival zone where the bucket was located. A session consisted of eight trials in which we measured the latency to eat in the bucket from the crossing departure line to the crossing arrival line. The animals were completely free to move, and the experimenter was out of sight during a trial, except to re‐lead the animal to the starting zone between trials. If an animal did not cross the arrival (or departure) line within five minutes, the experimenter would indicate the bucket by standing near it and calling the animal by name. A session was considered correct if the subject voluntarily entered the barn and reached the bucket in under 30 s in at least seven out of eight trials, and did not exhibit signs of distress (e.g., urinating or defecating more than once, or bleating excessively). The subject was considered ready to move to the next stage of the test when it had completed three consecutive correct sessions.

##### 1.2.1.2 Test

For the first repetition, the novel surface was a white plastic sheeting, and for the second one, it was a bright green mulching fabric. The novel surface was placed in the central zone (Figure A1). The procedure was the same as before: the animal voluntarily entered the starting zone, after which the experimenter closed the door and moved out of sight. The latency to eat from the bucket was recorded. The test ended either when the subject ate from the bucket or after five minutes if it did not eat.

#### 1.2.2 Surprise Test (S)

The sudden stimulus consisted of the opening of a dark blue umbrella. First, we had to familiarise the animals with the new, smaller test arena and the large grey screen. A fence separated a small zone (1 × 2 m), in which the experimenter could stand to open the umbrella during the test (the “umbrella zone”). We also had to teach the animals to position themselves correctly, facing the “umbrella zone”, to ensure they could see the umbrella during the test.

##### 1.2.2.1 Habituation

A bucket (the same as in the previous novel area test) containing crushed barley was placed on the ground, 3 m from the “umbrella zone”. The animal was then instructed to enter the test arena, in which no human experimenter was present. A session was considered correct if the subject ate from the bucket and faced the “umbrella zone” by itself within 30 s after entering the arena and showed no signs of distress. If the animal neither moved nor ate from the bucket within one minute, the experimenter entered the arena and indicated the bucket to the animal. The subject was considered ready to progress to the next stage of the test when it had completed three consecutive correct sessions.

##### 1.2.2.2 Test

On the day of the test, a human experimenter stood in the “umbrella zone” with the umbrella directed towards the bucket and prepared to open it. The bucket was empty. The experimenter waited for the animal to check the bucket, raise its head, and then opened and quickly closed the umbrella again. The test was filmed, and the videos were used to measure the subject's startle response.

#### 1.2.3 Novel Object (NO) Test

After the surprise test, we allowed the animals to re‐enter the test arena to familiarise themselves with the white lines that divided the arena into six equal sections.

##### 1.2.3.1 Habituation

As in the habituation for the surprise test, a bucket containing crushed barley was placed on the ground. We conducted at least two habituation sessions per individual. We considered a habituation session correct if the animal ate from the bucket in less than 30 s after entering the arena and did not show any particular reaction to the marks on the ground, nor signs of distress. After one successful habituation session, the animal could be tested.

##### 1.2.3.2 Test

An object, unknown to the sheep and goats, was placed at one end of the arena before the animal entered it. A different novel object was used for each repetition of the study. In the first session, this was a multicoloured, inflatable balloon in the shape of a birthday cake (0.5 × 0.3 × 1.2 m), and in the second session, it was a dark blue rubber ball with a diameter of 0.75 m. The test lasted three minutes, after which the experimenter opened the door and released the animal.

#### 1.2.4 Social Isolation (SI) Test

The day before the isolation test, we allowed the animals to enter the test arena so that they could see that the novel object was no longer there. The test consisted of isolating the animal from the main group, without the possibility of joining or seeing its conspecifics, and observing the reactions to isolation. It lasted three minutes during which the animals were filmed to record several behaviours (Table A3).

#### 1.2.5 Attraction Towards Conspecific: “Social Attraction Test” (SA)

This test consisted of isolating the test animal in the test arena, where the opportunity to join a familiar conspecific was available. The familiar conspecific was placed at one end of the test arena, behind a fence, in the “conspecific zone” (which was the same as the “umbrella zone” in the surprise test). Both individuals could then see, hear and smell each other. The aim of this test was to assess the reaction to a social attraction. The familiar conspecific was chosen according to the following criteria: (1) The conspecific had a strong relationship with the test animal. This was determined by analysing the social network: for each test animal, the conspecific was chosen from among the individuals with the most frequent links (i.e., a high link weight). (2) The difference in hierarchical rank between the test animal and its conspecific must not be too large; (3) The conspecific must voluntarily enter the zone. Among other behaviours, we assessed the latency of the test animal to join its conspecific, as well as the time it spent near the conspecific (Table A3). The test lasted three minutes.

#### 1.2.6 Separation From Conspecifics: “Social Retreat Test” (SR)

This test consisted of placing a familiar conspecific near the test arena and then removing it from the view of the test animal (without possibilities for the test animal to join it) in order to evaluate the response to social separation. The familiar conspecific was the same individual used in the attraction test for each test animal. It was placed in the “conspecific zone” for the first half of the test (i.e., 90 s). Then, without being seen by the test animal, the experimenter discreetly let the conspecific out by opening a side door. During the second half of the test, the test animal was alone. Four behavioural parameters (Table A3) were recorded throughout the test, and we calculated the difference between the behaviours expressed after and before the separation.

**TABLE A3:** Behavioural parameters recorded and further analysed for all personality tests (NA: Novel Area; NO: Novel Object; S: Surprise test; SI: Social Isolation; SA: Social Attraction; SR: Social Retreat). For the SR* test, the difference in behaviour before and after separation is calculated for each behaviour.

Behaviour	Test	Description	Type of recording
Locomotion	NO + SI + SA + SR*	Number of crossed zones per minute (to be considered in a zone, the subject must have all four legs in that zone)	Frequency
Vigilance	SI + SA + SR*	The animal is immobile, its head/neck upright and tense, and its ears forward for at least 1 s	Frequency
Vocalisations	NO + NA + SI + SA + SR*	Any kind of bleats made by the animal	Frequency
Sniff ground	SI + SA + SR*	Time spent where the animal sniffs the ground (with muzzle at 1‐10 cm from the ground)	Duration
Latency approach companion	SA	Latency to approach the conspecific (at least one part of the body less than 1 m)	Latency
Time close companion	SA	Time spent close to the conspecific (at least one part of the body less than 1 m)	Duration
Time contact companion	SA	Time spent interacting with the conspecific, i.e., touching or smelling (with muzzle at 1–10 cm from the conspecific)	Duration
Time in object proximity	NO	Time spent in the zone close to the object (less than 2 m from the object)	Duration
Time far object	NO	Time spent in the zone the furthest from the object (more than 4 m from the object)	Duration
Time interact	NO	Time spent interacting with the object, i.e., touching, smelling (with muzzle at 1–10 cm from the object)	Duration
Latency interact object	NO	Latency to touch or smell the object (with muzzle at 1–10 cm from the object)	Latency
Time in area proximity	NA	Time spent in the zone close to the surface (less than 50 cm from the surface)	Duration
Time start zone	NA	Time spent in the start zone	Duration
Time immobile	NA	Time spent immobile, with four legs on the floor	Duration
Latency to eat	NA	Differences in latencies to eat in the bucket in the last session of habituation	Latency
Latency interact area	NA	Latency to interact with the novel area, with any part of the body (leg, snout, with muzzle at 1–10 cm from the object)	Latency
Startle response	S	Magnitude of startle response. Code from 0 to 4: 0: no reaction 1: quivering 2: startle with legs, don't leave the ground 3: strong startle, and the subject steps backward for one or two steps 4: strong startle + flight (from more than two steps)	

#### 1.2.7 Statistical Analyses

All data were analysed using RStudio (version 2023.03). We performed principal components analyses (PCAs) for each test session to identify the relationships between the measured behaviours, using the *psych* package in R. We applied a Varimax rotation to the correlation matrix for each PCA as this is considered the most appropriate method for small sample sizes (Budaev 2010). PCAs are used to condense several correlated measures into a smaller number of principal components. The final number of principal components (PC) to be extracted can be determined using Cattell's scree test, Kaiser's criterion of eigenvalues > 1, Horn's Parallel test or Velicer's Minimum Average Partial (MAP) test (Graunke et al. 2013). We applied each of these methods to determine the optimal number of principal components for interpretation. The loadings of each measure on a principal component represent the correlation between the component and the measure in question, i.e., the loadings reflect the importance of each measure for the component. The principal components can be interpreted as different aspects of personality, i.e., personality traits. We used behavioural measures with loadings > 0.7 (“excellent”) and > 0.63 (“good”) within each PC as explanatory variables to identify the measured traits (Budaev 2010).

We performed a PCA on each species and each test session to identify groups of individuals exhibiting similar behaviours. We then performed a *k*‐means clustering analysis on the principal components from each PCA using the *stats* package in RStudio. We scaled and centred the individual scores on each component and determined the optimal number of clusters using different validation indices (cluster sums of squares, average silhouette, and gap statistics) calculated using the *factoextra* package. Finally, we calculated the cluster standardised means for each behavioural response in order to define the behavioural profiles of the different clusters. According to the majority of methods, the optimal number of clusters was three for each repetition in goats and two for each repetition in sheep. We examined whether the clusters produced for each session were defined by the same behavioural measures. Finally, we assessed the stability of each individual's personality over time by checking whether they belonged to the same cluster in the first and second repetitions.

#### 1.2.8 Personality Profiles in Goats

We retained three PCs that described 50.4% of the total variance from the PCA in the first test session of goats. The first PC explained 17.9% of the variance and comprised mainly behaviours from the novel area test. The second PC accounted for 16.4% of variance and was explained by measures of vocalisations in the social attraction and novel object tests, as well as measures of locomotion in the isolation and novel object tests and latency to approach the novel object. The third PC explained 16% of the variance and was most influenced by behaviours expressed in the social attraction test and vocalisations during the novel area test.

In the PCA from the second test session, we only retained two PCs that described 36.9% of the total variance. The first PC was similar to the first PC of the first repetition and explained 19.5% of the variance. The second PC accounted for 17.4% of the variance and was influenced by vocalisation measures in various contexts, as well as locomotion activity during social isolation.

Cluster analyses of these two PCAs produced three very similar clusters in each repetition. Individuals in cluster A were most inclined to approach and touch the novel area and the novel object. They vocalised less and were less vigilant in all the tests. They did not seek contact with conspecifics in the social attraction test and did not explore their environment, showing little locomotion during the novel object test and rarely sniffing the ground in different contexts. We describe these goats as “bold/calm”. Goats in cluster B vocalised the most during all tests, except the novel area test. They were the most active, demonstrating high levels of locomotion in the social isolation, attraction and novel object tests. However, they were the slowest to approach the novel object and did not spend time in proximity to the novel area, nor interact with the novel object. They did not seek contact during the social attraction test. We consider them to be “fearful/stressed”. Goats in the last cluster C were intermediate between the previous two groups. They were slow to approach and cross the novel area but spent a lot of time near it. They also sought contact with conspecifics during the social attraction test and spent time exploring their environment during the social isolation and social attraction tests. They were vigilant during separation and isolation from conspecifics. We describe these goats as “cautious/gregarious”.

Fourteen out of 20 goats belonged to the same cluster in the first and second test sessions. We kept the classification from the first session for further analysis.

#### 1.2.9 Personality Profiles in Sheep

We retained three principal components that described 54.7% of the total variance from the PCA on the first test session in sheep. The first PC explained 25.3% of the variance and was characterised by five behaviours measured in the novel area test. The second PC accounted for 15.8% and comprised four behaviours measured in the novel object test. Latency to approach the familiar conspecific during social attraction also loaded strongly on this dimension. The third PC explained 13.5% of the variance and was related to locomotion in social separation and social attraction. We also retained three PCs that explained 58.1% of the total variance in the PCA from the second test session. The first PC was similar to the first PC from the initial PCA, comprising mostly behaviours from the novel area test (24.3% of the total variance). The second PC accounted for 17.7% of the total variance and was described by measures of vocalisations in the social retreat, social isolation and novel object tests, as well as locomotion and exploration in the social retreat test. Finally, the third PC explained 16.1% of the total variance and resembled the second PC from the first test session as it was mostly influenced by reactions to the novel object test and by vigilance and proximity to conspecifics during the social attraction test.

Cluster analyses performed on each PCA for each repetition revealed two clusters in sheep, which remained consistent between the first and second test sessions. Sheep in cluster A were the fastest to approach the novel object and the novel area and spent time in proximity to them. They were the least vigilant in different situations, vocalised less, and did not startle when the umbrella opened. They displayed the greatest activity in the social attraction, isolation and retreat tests, as well as in the novel object test (high locomotion and exploration of their environment). We consider them to be “bold/active”. Individuals in cluster B were the opposite: they were slow to approach the novel area or the novel object and did not interact with or spend time near them. They were the most susceptible to startling during the surprise test. They vocalised the most and were the most vigilant in different situations. They displayed low levels of general activity (little sniffing and locomotion). We described them as “shy/gregarious”. The latency to approach conspecifics, proximity to conspecifics and contact with conspecifics during the social attraction test did not discriminate between the two clusters well. Seventeen out of 19 sheep belonged to the same cluster in the first and second repetitions. We used the classification from the first session for further analyses.

### 1.3 Details About Training

*First training step*: teaching subjects to discriminate between two distinct locations on the table and correctly point to one option.

The first training step was divided into two preliminary phases. The first phase consisted of training individuals to point at a food reward placed on the table and teaching them that food rewards could be either on the left or on the right side of the table. During a session, the reward was a slice of carrot. The experimenter initially placed it four times in the centre of the table, to encourage the subject to position themselves correctly in front of the grid and insert their snout to indicate the carrot. Once in position, the subject received the reward. For the 20 remaining trials, the subject first faced the middle of the table, and a carrot slice was then placed either to the right or to the left of the table, 10 times each in a semi‐randomised order. Once the subject moved to the correct side and maintained its position, it received the reward. A subject could not consume more than 24 carrot slices during a session. Each individual underwent as many sessions as necessary to learn to point unambiguously to the reward, i.e., to insert their snout through the grid and maintain the position without exhibiting “unwanted behaviours” such as attempting to go under or to the sides of the table, switching positions multiple times, or kicking or head‐butting the table. Once these criteria were met, the individual was considered ready to move up to the second training period. Note that some individuals added signals to their “pointing” such as licking towards the food (that they could not reach), scratching the floor with their leg, and gently bobbing their head towards the rewards.

The second phase focused on familiarising the animals with the task material and training them to distinguish between two distinct locations on the table, where only one was baited. The experimenter placed a single grey round plate on either the left or right side of the table and the opposite side had an empty plate. The two plates were subsequently covered with opaque over‐cups. The subject positioned themselves in the centre of the table, and then the experimenter simultaneously removed over‐cups, revealing the two options from which the subject had to choose. Each option was pseudo‐randomly baited, ensuring that the food reward was placed 10 times on each side during one session. The subject received the reward as soon as they correctly pointed to the baited side. If the subject pointed to the non‐baited side, the experimenter shifted the plate ahead of them to demonstrate its emptiness. The experimenter then redirected his/her attention to the baited side by tapping the table showing the rewards but without giving them to the subject. After pointing at the reward with at least 80% accuracy for two consecutive sessions (16 out of 20 correct trials), the subject had completed the training phase.

*Second training step*: discrimination of 1 vs 4 food rewards.

This training involved eight sessions per naïve individual, each consisting of 12 choices, with the side position of the larger quantity counterbalanced within a session and never presented more than three times in a row. The experimenter made no attempt to arrange the food items into a consistent pattern. All carrot pieces were uniform, as sliced into small pieces measuring 1 × 1 × 2 cm each (Figure A6). Some participants exhibited a side bias, and they entered a correction procedure. If a subject selected the reward (small or large) more than 10 times (out of 12) on the same side, in three consecutive sessions, we considered that they exhibited a side bias, leading us to move them to corrective sessions. Side bias correction involved two phases: first, we conducted two sessions of six choices in the same manner as the second training period, i.e., the subject had to choose between four carrot pieces and nothing, but the reward was positioned on the opposite side of their bias. Subsequently, the individual had to relearn to indicate the non‐preferred side. Second, we carried out four sessions consisting of 12 choices between the low‐quantity and the high‐quantity reward, with the high‐quantity reward consistently placed on the opposite side of the bias. The subject could return to testing once they had either achieved two consecutive successful sessions, i.e., more than 80% correct trials, or had successfully retrieved the reward every time during one session (100% success).

*Third training step (naïve and experienced animals)*: checking whether subjects prefer to choose the large quantity (1 vs. 4 and 1 vs. 8).

The last training step aimed to check whether all subjects (experienced and naïve individuals) would prefer the larger quantity when presented with the two following ratios: 1 versus 4 and 1 versus 8. Each ratio was presented equally during 12‐trial sessions, and for each, the larger quantity was presented as many times on the right as on the left, but never more than three times in a row on the same side. Subjects completed a minimum of two and a maximum of 12 sessions. Training stopped once the success criterion was reached, i.e., 10 out of 12 successes during two consecutive sessions (corresponding to a significant binomial test), or 100% success in one session.

### 1.4 Detailed Results

**TABLE A4:** Individual results. We show the percentage of successes (i.e., choosing the small reward) for each individual at different stages of the experiment (the first 20 sessions of the reversed‐reward contingency task and the corrective sessions, including the additional ones). We also indicate whether subjects exhibit a side preference by showing the percentage of choices made on the same side (left). The significance of success and side preference for each individual was established using a two‐tailed binomial test, with the probability of succeeding (or choosing either side) being 0.5. The significance level was set at 0.05. *EDE died before the end of the experiment and was therefore only tested in 144 trials (during RRC).

Subject	RRC (20 first sessions)		Corrective sessions (including additional corrective sessions)	
	Percentage of success in *N* = 240 trials	Side preference (% of selections of the left side in *N* = 240 trials)	Percentage of success	Side preference (% of selections of the left side)
BAL	40.8%, *p* = 0.005	88.3%, *p* < 0.001	50%, *N* = 48, ns	100%, *N* = 48, *p* < 0.001
CAN	21.3%, *p* < 0.001	39.6%, *p* = 0.001	47.9%, *N* = 48, ns	2%, *N* = 48, *p* < 0.001
DOM	38.8%, *p* < 0.001	88.8%, *p* < 0.001	45.8%, *N* = 48, ns	95.8%, *N* = 48, *p* < 0.001
LUN	40%, *p* = 0.002	86.7%, *p* < 0.001	47.9%, *N* = 48, ns	97.9%, *N* = 48, *p* < 0.001
POP	1.7%, *p* < 0.001	50%, ns	6.3%, *N* = 48, *p* < 0.001	43.8%, *N* = 48, ns
PRA	27.9%, *p* < 0.001	24.6%, *p* < 0.001	50%, *N* = 48, ns	100%, *N* = 48, *p* < 0.001
ROU	22.5%, *p* < 0.001	71.7%, *p* < 0.001	52.3%, *N* = 72, ns	95.8%, *N* = 72, *p* < 0.001
SOQ	10.8%, *p* < 0.001	48.3%, ns	25%, *N* = 48, *p* < 0.001	25%, *N* = 48, *p* < 0.001
ULK	45%, ns	6.3%, *p* < 0.001	50%, *N* = 48, ns	100%, *N* = 48, *p* < 0.001
EDE	18%, **N* = 144, *p* < 0.001	40.3%, **N* = 144, *p* = 0.02		
PEP	12.5%, *p* < 0.001	49.2%, ns	45.8%, *N* = 48, ns	4.2%, *N* = 48, *p* < 0.001
PET	39.2%, *p* < 0.001	89.2%, *p* < 0.001	50%, *N* = 48, ns	100%, *N* = 48, *p* < 0.001
RAS	4.2%, *p* < 0.001	50%, ns	29.2%, *N* = 48, *p* = 0.006	20.8%, *N* = 48, *p* < 0.001
TAG	30%, *p* < 0.001	76.7%, *p* < 0.001	63.3%, *N* = 60, *p* = 0.05	70%, *N* = 60, *p* = 0.003
TAR	43.8%, ns	92.9%, *p* < 0.001	70.8%, *N* = 24, ns	29.2%, *N* = 24, ns
TYR	60.4%, *p* = 0.001	81.3%, *p* < 0.001		
VIR	38.8%, *p* < 0.001	86.3%, *p* < 0.001		
VOL	41.3%, *p* = 0.008	89.6%, *p* < 0.001	50%, *N* = 48, ns	100%, *N* = 48, *p* < 0.001

#### 1.4.1 GLMM1

With GLMM1, we examined factors likely to influence success in the RRC task. We used a generalised linear mixed model with a binomial distribution and a logit link function. We included the following as fixed factors: species, ratio, age, experience, session number and any interaction between species and the other predictors. We included subject identity as a random factor.

*Assumptions checked included*: uniform distribution of residuals (Kolmogorov–Smirnov test: *D* = 0.01, *p*‐value = 0.72); no over‐ or under‐dispersion (Dispersion test: dispersion = 1.03, *p*‐value = 0.58); no outlier (Outlier test: *p*‐value = 0.40) and VIF values less than three (Daoud 2017).

**TABLE A5:** GLMM 1 analysis of variance that shows the contribution of each factor.

Variables	*χ* ^2^	df	*p*
Species	2.8	1	0.09
**Age**	**5.9**	**2**	**0.05**
Ratio	2.4	1	0.1
**Session**	**121.5**	**1**	**< 0.001**
Experience	0.07	1	0.8
Species × Age	1.6	2	0.4
Species × Ratio	1.2	1	0.3
Species × Session	1.0	1	0.3
Species × Experience	1.6	1	0.2

*Note:* Bold values indicate statistical significance with *p*‐values below 0.05.

**TABLE A6:** Post hoc analysis of contrasts (with Tukey correction) from GLMM1.

		Contrast	Estimate	SE	*z*. ratio	*p*
Age	**Goats**	**Young–adult**	**1.6**	**0.7**	**2.4**	**0.04**
		Young–aged	0.9	0.9	0.9	0.6
		Adult–aged	−0.8	0.9	−0.8	0.7
	Sheep	Young–adult	0.4	0.8	0.5	0.9
		Young–aged	−0.04	0.7	−0.06	1
		Adult–aged	−0.4	0.7	−0.6	0.8
Experience	Goats	Experienced–naïve	0.2	0.8	0.3	0.8
	Sheep	Experienced–naïve	−1.0	0.6	−1.7	0.09
Ratio	Goats	1:8–1:4	−0.2	0.1	−1.6	0.1
	**Sheep**	**1:8–1:4**	**−0.3**	**0.1**	**−3.1**	**0.002**

*Note:* Bold values indicate statistical significance with *p*‐values below 0.05.

#### 1.4.2 Effect of Personality Profiles on Success

We conducted non‐parametric tests for each species in order to examine the effect of personality (i.e., behavioural profiles) on success (i.e., percentage of success in the first 20 sessions). We found no significant differences between personality profiles on success in the RRC test in goats (Kruskal‐Wallis: *K* = 3.1, df = 2, *p* = 0.2), nor in sheep (Mann–Whitney: *N*_1_ = 4, *N*_2_ = 4, *U* = 12, *p* = 0.3).

#### 1.4.3 GLMM2

With GLMM2, we examined how side bias developed during the test. We used a generalised linear mixed model with a binomial distribution and a logit link function. We included species and session as predictors, as well as the interaction between the two, and added subject identity as a random factor.

*Assumptions checked included*: uniform distribution of residuals (Kolmogorov–Smirnov test: *D* = 0.05, *p*‐value = 0.32); no over‐ or under‐dispersion (Dispersion test: dispersion = 1.12, *p* = 0.24); no outlier (Outlier test: *p* = 1); and VIF values less than three.

**TABLE A7:** GLMM 2 analysis of variance to analyse the contribution of each factor.

Variables	*χ* ^2^	df	*p*
Species	**7.6**	**1**	**0.006**
Session	**54.6**	**1**	**< 0.001**
Species × Session	**16.3**	**1**	**< 0.001**

*Note:* Bold values indicate statistical significance with *p*‐values below 0.05.

*Post hoc analysis (with Tukey correction) from GLMM2*: The analysis of session trends revealed a significant interaction between species and session, with sheep showing a steeper increase in success rate over sessions compared to goats (contrast: estimate = −1.2, SE = 0.3, *p* < 0.001). Specifically, while goats exhibited a modest positive trend (trend = 0.3, SE = 0.04, lower‐CI = 0.2, upper‐CI = 0.4), sheep showed a markedly stronger improvement over time (trend = 1.5, SE = 0.3, lower‐CI = 0.9, upper‐CI = 2.1).
